# Does Utilitarian Policy such as Smoking Cessation Lend Support to Wider Aspirin Use?

**DOI:** 10.3934/publichealth.2015.2.223

**Published:** 2015-06-11

**Authors:** Gareth Morgan

**Affiliations:** Cochrane Institute, Cardiff University, Cardiff, Wales, UK CF14 4XN

**Keywords:** aspirin, policy, vascular disease, cancer, gastrointestinal effects, smoking, public health, utilitarian

## Abstract

Tobacco control policy seems to be based on a utilitarian principle that public health is best served by a range of measures that will provide overall population benefit. Aspirin may have a potential wider role since meta-analysis of randomized controlled trials shows it reduces the risk of a first vascular event and also cancer. Are smoking cessation and the public health potential of aspirin different? The benefit versus risk balance of aspirin, an inexpensive and easily available medicine, deserves serious consideration as a public health measure in middle age. Smoking cessation and wider aspirin use are not seen as either competing or duplicating policy areas, but complementary. Their comparison has been purposefully selected because of common impacts, namely reduced vascular disease and cancer with increases in undesirable effects, notably gastrointestinal pathology. Part of the driver for this paper is to convey the message that public health policy has benefits and risks and the concept of a universally effective policy is unrealistic. Is it time for public health action to increase the use of aspirin?

The World Health Organisation (WHO) recognizes the morbidity and mortality associated with smoking, including an increased risk of vascular events and cancer, as per the website link provided at the end of this commentary. Public health policy efforts to reduce smoking in the population are broad, intensive and sustained. One of the drivers for these efforts, which range from advertising bans to helping smokers quit, is to improve overall population health. The health risks of passive smoking are a further driver, with some countries now banning the use of tobacco products in public places. Whilst the public health view supported by WHO is that smoking is to discouraged, there are some points of controversy. For example, some smokers may feel disenfranchised, stigmatized and concerned that cessation policy compromises their freedom of choice. Furthermore, there may be some adverse health aspects of smoking cessation, such as weight gain [1] and the exacerbation of the gastrointestinal condition ulcerative colitis [2].

Thus, the situation is not as clear cut as many public health professionals might believe and there are also complex vested interests, not least by the tobacco industry and also among companies with marketed smoking cessation products. A further consideration in this complex situation is that taxation from smoking can be a major contributor to gross domestic product whilst some pressure groups campaign for the freedom to enjoy their consumption of tobacco products. Hence policy is complex and needs to balance many competing views, of which health is only one.

Tobacco control policy seems to be based on a utilitarian principle that public health is best served by a range of measures that will provide overall population benefit. In achieving the benefits of reduced incidence of vascular events and cancer, some members within the target cohort will experience undesirable effects, namely inconvenience, psychological distress and exacerbation of a gastrointestinal condition. This utilitarian trade-off between benefits versus harms is also present in other public health situations, such as breast cancer screening [3]. How might the benefit versus harm trade-off inform the wider use of aspirin in the population?

To qualify this paper, smoking cessation and wider aspirin use are not seen as either competing or duplicating policy areas, but complementary. Their comparison has been purposefully selected because of common impacts, namely reduced vascular disease and cancer with increases in undesirable effects, notably gastrointestinal pathology. Part of the driver for this paper is to convey the message that public health policy has benefits and risks and the concept of a universally effective policy is unrealistic.

Aspirin use for the secondary prophylaxis of vascular events is a mainstream part of clinical practice. There are issues that remain to be addressed with aspirin and secondary prophylaxis, namely non-compliance. As a wider public health issue, aspirin may have a potential wider role since meta-analysis of randomized controlled trials shows it reduces the risk of a first vascular event [4] and also cancer [5]. The former benefit is small, perhaps 10% in a low risk population, while the latter benefit may take about 10 years to be observed. Of course aspirin also increases the risk of internal bleeding, usually in the gastrointestinal organs, although meta-analysis of randomized controlled trials suggest that this bleeding is usually minor, with no evidence of excess mortality [6]. There is an additional complexity with this situation and it also needs to be noted that aspirin leads to fewer ischemic strokes but an excess of hemorrhagic strokes occur. The benefit-risk trade off with aspirin is complex and cannot be judged entirely on the numbers of events avoided or caused, since the seriousness of the respective conditions is markedly different. For example, how can a cancer be equated to a gastrointestinal bleed when the former carries a much higher mortality rate than the latter?

The potential of aspirin to be taken at about the age of 50 years for the prophylaxis of vascular events and cancer is a matter of debate [7],[8], with respect to healthy ageing policy. However, are smoking cessation and wider aspirin use different? The former removes an overall harm with benefits to some people and the latter promotes an overall benefit with harms to some people. Also, their underpinning principles are the same, namely that the public health overall will be improved. Furthermore, there are two other drivers for the wider use of aspirin. The first is that media reports are already bringing the benefits and risks of aspirin into the public domain. A related point is that self-medication of aspirin in people aged 50 years and above is significant, with one survey showing community use of 30% [9]. Secondly, evidence from a Citizen's Jury suggests there is an appetite in the general public to make informed choices about their own lifestyle, of which aspirin may play a role [10]. This may bring some tensions between a medical model of illness and social model of wellbeing.

The benefit versus risk balance of aspirin, an inexpensive and easily available medicine, deserves serious consideration as a public health measure. Whilst opposition to the wider use of aspirin might remain, the parallels to the principles underpinning smoking cessation offer a further framework for consideration. Both wider aspirin use and smoking cessation would lead to a reduction in the morbidity and mortality from vascular disease and malignancy, with some excess gastrointestinal pathology observed. Further, people who decide to take an aspirin and smokers who either decide to continue or stop are exercising personal choice. Should public health help decision making in both situations be an informed one?

Opposition to the wider use of aspirin may have more to do with risk aversion, perhaps motivated by non-maleficence, or a rigid adherence to a medical model in which ‘doctors decide’ [11]. These points of opposition are open to challenge on the basis of evidence collected on the behaviors [9] and expectations [10] of the public. It is therefore timely, perhaps even overdue, for policy to be developed to convert the public health potential of aspirin into a reality. Continued inaction on this would only bring into question the leadership and priorities within the public health profession. Further, inaction on aspirin may be as unethical as doing nothing on tobacco control.

The limits to this paper are acknowledged, including no analysis on secondary or tertiary effects, how policy could be developed or what the unintended effects of wider aspirin use could be. These fall outside of the scope of this paper, which is intended only to convey general principles and start a wider discussion. So what happens next? If in the name of the public good, interventions with known harms, such as smoking cessation and breast cancer screening, are to continue then for consistency purposes, it is timely for aspirin use in middle age to be considered as part of healthy ageing policy.

Public health has played a major role in identifying the risks of smoking and influencing policy. If public health fails to take action to promote the wider use of aspirin, do we conclude that we now lack the necessary courage and leadership to progress challenging policy?
